# Exploring the wound-healing potential and seasonal chemical variability of the Formosan Callery pear *Pyrus calleryana*: implications for therapeutic applications

**DOI:** 10.1080/13880209.2024.2378011

**Published:** 2024-07-26

**Authors:** Bo-Rong Peng, Xin-Yun Tang, Yun-Shiuan Chen, Kuei-Hung Lai, Mei-Hsien Lee

**Affiliations:** a Graduate Institute of Pharmacognosy, College of Pharmacy, Taipei Medical University, Taipei, Taiwan; b PhD Program in Clinical Drug Development of Herbal Medicine, College of Pharmacy, Taipei Medical University, Taipei, Taiwan; c Center for Reproductive Medicine and Sciences, Taipei Medical University Hospital, Taipei, Taiwan

**Keywords:** LC-MS/MS, GNPS, *MMP-1*, *COL1A1*

## Abstract

**Context:**

*Pyrus calleryana* Decne (Rosaceae), renowned for its therapeutic properties, is known to moisturize the lungs (removing dryness; relieving cough), clear heat (acting as an antipyretic; febrifuge) and aid in detoxification (relieving pyogenic inflammation; eliminating toxins). However, scientific evidence supporting its efficacy in wound healing is lacking.

**Objective:**

This study investigated *P.* *calleryana* samples collected over a year to explore metabolite variations and their impact on skin wound-healing activities.

**Materials and methods:**

*P. calleryana* (PC) twigs and leaves were collected from the Matsu Islands, Taiwan, spanning 2018–2020. Extracts were prepared using 95% ethanol or water, and we assessed the chemical composition, total phenolic/triterpenoid contents and antioxidant properties. Metabolites were analysed via LC–MS/MS and molecular networking. Wound healing potential was evaluated on WS-1 cells through MTT and migration assays, and gene expression analyses, with tests including control (DMSO), compounds **1** (3′-hydroxylbenzyl-4-hydroxybenzoate-4′-*O*-β-glucopyranoside) and **2** (vanilloylcalleryanin) (100 µM), and a positive control (ascorbic acid, 100 µM) for 24 h.

**Results:**

Significant variations in extract compositions were observed based on the solvent used, with distinct metabolomic profiles in extracts collected during different months. Notably, compounds **1** and **2** showed no cytotoxic effects on human dermal fibroblast cells and significantly accelerated wound closure at 100 μM. A gene expression analysis indicated upregulation of wound healing-associated genes, including *MMP-1* (matrix metalloproteinase-1) and *COL1A1* (collagen, type 1, alpha 1).

**Conclusions:**

This study reports the first evidence of PC compounds aiding wound healing. Utilizing Global Natural Products Social Molecular Networking (GNPS) and principal component analysis (PCA) approaches, we unveiled metabolomic profiles, suggesting the potential to expedite wound-healing.

## Introduction

Human skin is equipped with several remarkable protective mechanisms that play vital roles in safeguarding the body from various external threats and maintaining overall health. These mechanisms work together to create barriers that shield the internal organs, tissues and systems from potential harm, including a physical barrier, chemical barrier and immunological barrier. The skin also produces sweat, sebum, melanin, sensory melanocytes, hair and nails, and heals wounds (Lai-Cheong and McGrath 2017; Walters and Roberts 2002).

The wound-healing process is a complex series of events that the body initiates to repair damaged tissues and restore the skin’s integrity and functionality. It is a natural and essential response to injury or trauma. The wound-healing process can be broadly divided into three main phases: an inflammatory phase, a proliferative phase and a remodelling phase (Landén et al. 2016).

During the proliferative phase of wound healing, the initial wound matrix formed during haemostasis is gradually replaced by granulation tissue. This specialized tissue is abundant in fibroblasts, granulocytes, macrophages and blood vessels, which are all intertwined with collagen bundles. Granulation tissue serves a vital role in restoring both structure and function to injured skin (Young and McNaught 2011; Cheng et al. 2016). Fibroblasts, originating from fibrocytes in prolonged wounds, contribute to tissue repair, promote angiogenesis and release matrix metalloproteinases (MMPs) to break down the matrix. They also deposit collagen and extracellular matrix (ECM) components (Landén et al. 2016; Tracy et al. 2016).

MMPs are a family of zinc-containing endopeptidases that can break down protein components of the ECM. There are at least 23 MMPs expressed in human tissues, and they are responsible for cell proliferation, migration and differentiation. MMPs are classified into six groups: collagenases, gelatinases, stromelysins, matrilysins, membrane-type MMPs and remaining MMPs (Cui et al. 2017). The group of collagenases, including *MMP-1* (matrix metalloproteinase-1), MMP-8 and MMP-13, is triggered during wound healing (Caley et al. 2015). MMP-1, also called collagenase-1, was observed to be secreted by fibroblasts during the formation of granulation tissue and angiogenesis in the early reepithelialization phase, which might promote cell migration while cleaving type I collagen for collagen turnover (Trung et al. 2016; Mathew-Steiner et al. 2021). Type I collagen is the main component of the ECM in skin. It is composed of two α1 (I) peptide chains and one α2 (I) peptide chain, respectively, encoding collagen, type 1, α1 (*COL1A1*) and *COL1A2* (Stefanovic 2013). The *MMP-1* gene encodes an enzyme that breaks down collagen, and the *COL1A1* gene produces collagen; therefore, both are crucial for wound healing, and balancing their roles is vital for effective tissue repair (Almeida et al. 2015).

*Pyrus* is a large genus belonging to the Rosaceae family, with nearly 70 species in Europe to East Asia, southward to North Africa and the Himalayas; and only two are native to Taiwan: *P. calleryana* (PC) Decne. and *P. taiwanensis* Iketani & H. Ohashi (Ohashi 1993). In traditional Chinese medicine, the roots, stems, leaves and fruit of Callery pear PC are all utilized to treat coughs, acute conjunctivitis, stomach pain, indigestion, dysentery, etc. (Teng et al. 2020). In traditional Chinese folk usage, PC is used to treat scabies and skin tinea. The method involves taking the root bark, boiling it in water to extract the juice, and using it to cleanse the affected area (Lu and Ren 2021). The previous literature reported that some *Pyrus* species were found to have different medicinal effects, such as anticancer, antiviral, laxative, anti-inflammatory, antipyretic, antimicrobial, antioxidant and mild estrogenic activities (Nassar et al. 2011; Ibrahim and Hammoudi 2020). Chemically, PC contains flavonoids (from leaves) (Challice and Williams 1968), phenolic acid (from leaves and fruits) (Nassar et al. 2011; Yim and Nam 2016), phenolic esters (from the fruit) (Nassar et al. 2011), phenolic glycosides (from leaves) (Challice and Williams 1968) and triterpenoids (from the stem bark) (El-Hawary et al. 2003). In this investigation, recognizing that harvesting root bark can potentially harm the plant’s well-being and raise ecological concerns, we prioritized sustainable supply and environmental friendliness. Given these considerations, our study centred on examining the effects of PC’s twigs and leaves and their isolated compounds on wound healing. The chemical constituents of PC extracts from different months were also analysed by liquid chromatographic–tandem mass spectrometric (LC–MS/MS) and Global Natural Products Social Molecular Networking (GNPS) tools.

## Materials and methods

### Reagents

Acetic acid (J.T. Baker, Phillipsburg, NJ), acetonitrile (ACN) (LC Grade, Merck, Darmstadt, Germany), ascorbic acid (Sigma-Aldrich, St. Louis, MO), 3-(4,5-dimethylthiazol-2-yl)-2,5-diphenyltetrazolium bromide (MTT) (Sigma-Aldrich, St. Louis, MO), dimethyl sulphoxide (DMSO) (J-T Baker, Phillipsburg, NJ), 1,1-diphenyl-2-picrylhydrazyl (DPPH) (Sigma-Aldrich, St. Louis, MO), Dulbecco’s phosphate-buffered saline (PBS) (Gibco, Waltham, MA), ethanol (EtOH) (ACS Grade, Echo Chemical, Miaoli, Taiwan), Folin–Ciocalteu’s phenol reagent (J-T Baker, Phillipsburg, NJ), formic acid (FA) (Riedel-de Haën, Seelze, Germany), gallic acid (Sigma-Aldrich, St. Louis, MO), hydrochloric acid (HCl) (Riedel-de Haën, Seelze, Germany), methanol (MeOH) (ACS Grade, Echo Chemical, Miaoli, Taiwan), MeOH (LC Grade, Merck, Darmstadt, Germany), oleanolic acid hydrate (TCI, Tokyo, Japan), perchloric acid (HClO_4_) (Sigma-Aldrich, St. Louis, MO), sodium carbonate (Na_2_CO_3_) (Sigma-Aldrich, St. Louis, MO), sodium hydroxide (NaOH) (Honeywell, Muskegon, MI), Trypan blue (Sigma-Aldrich, St. Louis, MO), trypsin (Gibco, Waltham, MA), vanillin (Sigma-Aldrich, St. Louis, MO), minimum essential medium (MEM) (Gibco, Waltham, MA), sodium bicarbonate (NaHCO_3_) (Mallinckrodt, St. Louis, MO), sodium pyruvate (Gibco, Waltham, MA), non-essential amino acids (Gibco, Waltham, MA) and foetal bovine serum (FBS) (Gibco, Waltham, MA). Other chemicals were of the highest grade commercially available.

### Plant materials and extraction

Twigs and leaves from the same PC tree (26.153459°N; 119.916440°E) were collected (ca. 100–300 g) monthly from the Matsu Islands, Taiwan, in 2015 (voucher no. PC-S-2015) and during the years 2018–2020 (voucher no.: PC-S-2018∼PC-S-2020). A voucher specimen bearing the numbers PC-S-2015 and PC-S-2018 ∼ 2020 was stored in the Taipei Medical University. Dr. Chi-Luan Wen, from the Taiwan Seed Improvement and Propagation Station (TSIPS), was identified and verified the plant species. Dry twigs and leaves were immersed and extracted twice with a 10-fold volume of 95% EtOH or water. The solvent was subsequently removed using vacuum pumps, and the total phenolic content, total triterpenoid content, and DPPH radical-scavenging ability of these extracts were then assessed. We calculated the yields of plant extracts using the following formula: yield (%) = (mass of the extract/mass of the dried raw plant material) × 100%.

### Compound isolation

**Figure 1.**
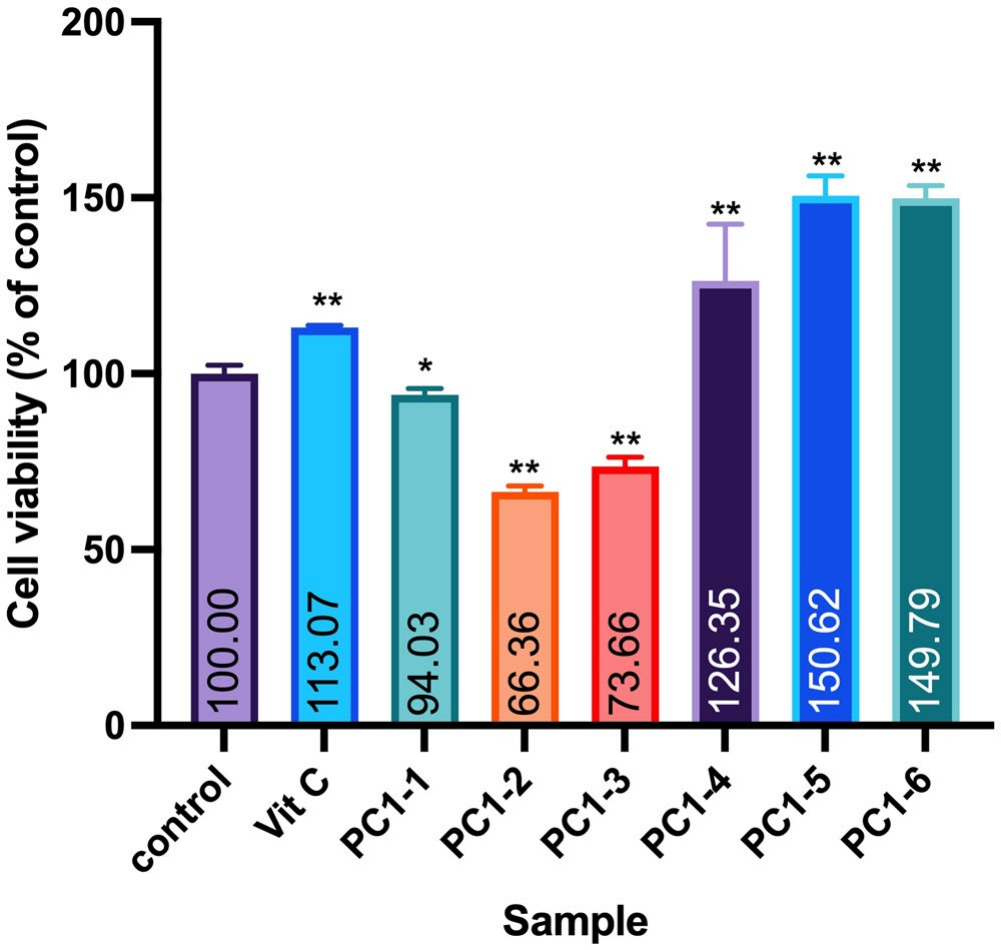
Effect of PC95E fractions on cell viability of WS-1 cells. WS-1 cells were seeded in a 48-well plate and treated with PC95E fractions (50 μg/mL) for 24 h. Vitamin C (ascorbic acid, 50 μM) was used as a positive control. Cell viability was measured by a MTT assay. Results are expressed as a ratio relative to the control. Each determination was performed in triplicate, and values are presented as mean ± standard deviation. *p < .05 and **p < .01 vs. the control group.

Twigs of PC were collected in August 2015 from Matsu Islands in Taiwan (GPS: 26.153459°N, 119.916440°E). Air-dried and sliced plant materials (6 kg) were infiltrated and extracted twice with 95% EtOH at room temperature. A sticky residue (190 g) was obtained after the organic solvent was removed on a rotary evaporator at 35 °C under reduced pressure. Extracts were fractionated into six portions (PC1-1 to PC1-6) with a Diaion^®^ HP-20 resin column using water, 20% MeOH, 40% MeOH, 60% MeOH, 80% MeOH and pure MeOH as eluents. Cell viability testing against the WS-1 cell line was conducted with all fractions. Following bioactivity guidelines, fraction PC1–4 was selected for compound separation due to its non-cytotoxicity (Figure 1). The PC1–4 portion (14.7 g) was subjected to C-18 silica gel column chromatography (CC) and yielded 15 fractions, A–O, when eluted with water–MeOH (1:0 → 0:1, v/v). Fraction H (200 mg) was separated into three subfractions (H1–H3) through semipreparative high-performance liquid chromatography (HPLC) using 45% MeOH (at a flow rate of 2 mL/min) as the eluent. Subfraction H3 (59 mg) was further purified by semipreparative HPLC (MeOH–H_2_O, 35:65, v/v, 2 mL/min) to provide compounds **1** (12 mg) and **2** (32 mg). Compounds **1** and **2** were identified as 3′-hydroxybenzyl-4-hydroxybenzoate-4′-*O*-β-glucopyranoside (**1**) (Nassar et al. 2011) and vanilloylcalleryanin (**2**) (Challice and Williams 1968) through analyses of their MS and nuclear magnetic resonance (NMR) spectroscopic data.

3′-Hydroxybenzyl-4-hydroxybenzoate-4′-*O*-β-glucopyranoside (**1**). White powder; UV (MeOH) *λ*_max_ (log *ε*): 258 (4.10) nm; ESIMS at *m/z* 421.11 [M − H]^−^; ^1^H NMR (500 MHz, DMSO-*d*_6_) *δ*_H_: 7.82 (2H, d, *J* = 8.8 Hz, H-2, H-6), 7.10 (1H, d, *J* = 8.3 Hz, H-5′), 6.89 (1H, d, *J* = 2.0 Hz, H-2′), 6.84 (2H, d, *J* = 8.8 Hz, H-3, H-5), 6.81 (1H, dd, *J* = 8.3, 2.0 Hz, H-6′), 5.15 (2H, s, H-7′), 4.68 (1H, d, *J* = 7.4 Hz, H-1″), 3.73–3.11 (5H, m, H-2″–H-6″).^13^C NMR (125 MHz, DMSO-*d*_6_) *δ*_C_: 165.4 (C-7), 162.1 (C-4), 146.7 (C-3′), 145.2 (C-4′), 131.5 (C-2, 6), 131.0 (C-1′), 120.3 (C-1), 119.2 (C-6′), 116.6 (C-5′), 115.7 (C-2′), 115.4 (C-3, 5), 102.2 (C-1″), 77.2 (C-5″), 75.9 (C-3″), 73.3 (C-2″), 69.8 (C-4″), 65.4 (C-7′), 60.8 (C-6″).

Vanilloylcalleryanin (**2**). White powder; UV (MeOH) *λ*_max_ (log *ε*): 264 (4.05), 219 (4.38) nm; ESIMS at *m/z* 451.12 [M − H]^−^; ^1^H NMR (500 MHz, CD_3_OD) *δ*_H_: 7.57 (1H, dd, *J* = 8.2, 2.0 Hz, H-6), 7.55 (1H, d, *J* = 2.0 Hz, H-2), 7.19 (1H, d, *J* = 8.3 Hz, H-5′), 6.95 (1H, d, *J* = 2.1 Hz, H-2′), 6.87 (1H, dd, *J* = 8.3, 2.1 Hz, H-6′), 6.84 (1H, d, *J* = 8.2 Hz, H-5), 5.21 (2H, s, H-7′), 4.78 (1H, d, *J* = 7.4 Hz, H-1″), 3.72 (1H, br s, H-6″), 3.88 (3H, s, 3-OCH_3_), 3.71 (1H, br s, H-6″), 3.48 (1H, m, H-2″), 3.46 (1H, m, H-3″), 3.41 (1H, m, H-5″), 3.40 (1H, m, H-4″). ^13^C NMR (125 MHz, CD_3_OD) *δ*_C_: 168.1 (C-7), 122.6 (C-1), 149.0 (C-3), 148.6 (C-3′), 146.9 (C-4′), 133.5 (C-1′), 121.0 (C-6), 153.3 (C-4), 121.0 (C-6′), 118.9 (C-5), 117.2 (C-2), 104.4 (C-1″), 78.5 (C-5″), 77.7 (C-3″), 75.0 (C-2″), 71.4 (C-4″), 67.3 (C-7′), 62.6 (C-6″) and 56.6 (3-OCH_3_).

### Determination of total phenolic contents

The total phenolic content of each extract was determined by a modified Folin–Ciocalteu method. The sample solution was mixed with an equal volume of 1 N Folin–Ciocalteu’s reagent – 20% Na_2_CO_3_. After a 25 min incubation period at room temperature, the reaction mixture was centrifuged at 5000 rpm for 10 min. The supernatant was measured at 730 nm on a spectrophotometer. The total phenolic content was expressed as gallic acid equivalents (GAEs) in mg/g of dry plant extract (Chang and Lin 2012).

### Determination of total triterpenoid contents

The total triterpene content was measured using a vanillin–HClO_4_ assay method, with some modifications. Briefly, 0.5 mL of each of the extracts or the standard oleanolic acid solution was lyophilized and mixed with 0.2 mL of a vanillin–acetic acid solution (5:95, w/v) and 1.0 mL HClO_4_. After incubation for 10 min in a 60 °C water bath, the mixture was chilled to room temperature, and 5 mL of acetic acid was added. It was then allowed to sit at room temperature for 15 min. The absorbance was determined at 548 nm with a blank solution as a reference. Results are expressed as milligrams of oleanolic acid equivalents (OAEs)/g of sample (Kou et al. 2015).

### Assay for DPPH-scavenging activity *in vitro*

Each individual test preparation underwent a reaction with DPPH in a MeOH solution. After incubation for 20 min at room temperature in the dark, the absorbance was read at 517 nm as previously described (Fitriana et al. 2018).

### Ultra-performance LC–MS/MS conditions for non-targeted fragment ion collection

MS/MS data were collected based on a Waters SYNAPT G2 LC/quadrupole time of flight (Q-TOF; Waters, Milford, MA) system. Chromatographic separation before the MS spectrum was performed using a C18 column of Waters Acquity UPLC BEH (Waters, Milford, MA, 1.7 μm, 2.1 × 100 mm). The mobile phase was set to MeCN (A, containing 0.1% FA)/water (W, containing 0.1% FA) gradient sequences: 0.01–7 min, 5–10% A; 7–15 min, 10–50% A; 15–17.5 min, 50–60% A; 17.5–25 min, 60% A; 25–27.5 min, 60–70% A; 27.5–28.5 min, 70–100% A; and 28.5–30 min, 100% A. The flow rate was set to 0.5 mL/min, and the temperature of the column part was maintained at 40 °C in an oven. The extract (4 mg) was dissolved in 1 mL of MeOH (5000 ppm) and was filtered using a 0.22-mm membrane filter. A sample was automatically injected with an automatic syringe with a 5-mL volume per injection. Non-targeted MS and MS/MS data were collected within the range of *m/z* 100–2000. An automated data-dependent acquisition (DDA) approach was applied for the MS/MS scans, and non-targeted selections of five precursor ions were fragmented with ramping of the collision energy from 10 to 50 eV. The acquired MS data were finalized by Waters MassFragment software (MassLynx4.1, Waters, Milford, MA) (Wang et al. 2023).

### The global natural product social (GNPS)-based molecular networking (MN) analysis

A GNPS web-based platform (https://gnps.ucsd.edu) was applied to analyse the output of the MS/MS MN data. MS/MS spectra were window-filtered according to the top five strongest ion peaks in the ±50-Da window throughout the spectrum. A network was then created in which linkages between nodes were filtered by a cosine value above 0.70 and at least four matched peaks. Nodes that appeared in the network were annotated based on the experimental MS/MS fragmentation of the isolates. The molecular network was visualized and laid out using Cytoscape 3.8.2 (National Resource for Network Biology (NRNB), San Diego, CA) (Wang et al. 2023).

### Cell culture

A normal human dermal fibroblast WS-1 (CVCL_2766, CRL-1502) cell line was purchased from the Bioresource Collection and Research Center (Hsinchu, Taiwan). The cell line was kept in a 5% CO_2_ humidified atmosphere at 37 °C. Minimum essential medium was the growing medium for the WS-1 cell line. Glutamine (2 mM), antibiotics (100 µg/mL streptomycin and 50 units/mL penicillin) and FBS (10%) were used to supplement the growth medium (Huang et al. 2017).

### Cell viability assay

Cell culture plates were used for the MTT assay (thiazolyl blue tetrazolium bromide, Sigma-M2128, St. Louis, MO). Cells were seeded at 4 × 10^4^ cells in 500 µL medium and plated in each well of a 48-well. When the cell density reached 60–70% confluence, the medium was replaced by drug-containing media. The cells were treated with either DMSO (control, 0.1%), fractions (50 μg/mL), compounds (100 µM, dissolved in DMSO) or a positive control (ascorbic acid, 100 μM, dissolved in DMSO), and cultures were incubated for an additional 24 h. For the MTT assay, the medium was replaced by 0.5 mg/mL MTT medium and was incubated for 4 h at 37 °C. The medium was then removed and the purple crystals in cells were dissolved by 200 μL DMSO followed by 10 min incubation, then transferred 100 μL of aliquot of the lysate to a 96-well plate. The relative cell viability was determined by the light absorbance at 600 nm (Huang et al. 2017).

### Wound-scratch test assay

WS-1 cells were seeded in 48-well plates, cultured in fibroblast medium containing 10% FBS, and grown to confluent cell monolayers. The medium was pipetted out and discarded; a small area was scratched using a pipette tip, and cells were rinsed with PBS to remove loose cell debris. Fibroblast media with either DMSO (control, 0.1%), compounds (60, 80 or 100 µM, dissolved in DMSO) or the positive control (ascorbic acid, 100 μM, dissolved in DMSO) were replaced, and the plates were incubated at 37 °C with 5% CO_2_. The distance between the two edges of cells which were scratched by the pipette tip was then inspected microscopically at 24 h. As WS-1 cells migrated to fill in the scratched area, images were captured with a digital camera attached to a microscope and computer system. Experiments were performed in triplicate, and data were analysed with ImageJ software (Bethesda, MD) (Muhammad et al. 2013).

The percent wound closure was calculated using the following formula:

$$\begin{array}{l} \text{Wound closure }\left(\text{\%}\right)=\\ \frac{\text{wound area at initial time }\left(t_{0}\right)-\text{wound area at }24\text{ h time }\left(t_{24}\right)}{\text{wound area at initial time }\left(t_{0}\right)}\times 100 \end{array}$$

### Real-time quantitative polymerase chain reaction (qPCR)

WS-1 cells were treated with MEM containing DMSO (control, 0.1%), compounds (60, 80 or 100 µM, dissolved in DMSO) or the positive control (ascorbic acid, 100 μM, dissolved in DMSO) for one day in 6 cm dishes. Medium was replaced with PBS for rinsing dishes. Total messenger (m) RNA was extracted by the following process. The WS-1 cell line was extracted with the TRIzol reagent/chloroform in 5:1 ratio and was incubated at room temperature for 3 min. Following centrifugation (12,000 × *g*, 15 min, 4 °C), 0.5 mL of isopropanol (per 1 mL of the TRIzol reagent) was used to precipitate RNA from the upper aqueous phase by pipetting gently, then was incubated at room temperature for 10 min. The RNA pellet formed after centrifuged at 12,000 × *g* for 10 min at 4 °C. The RNA pellet was washed with 1 mL of 75% EtOH (per 1 mL of the TRIzol reagent), then centrifuged at 7500 × *g* for 5 min at 4 °C, air dried the RNA pellet, then resuspended the pellets in nuclease-free water. The High Capacity cDNA Reverse Transcription Kit (Thermo, Waltham, MA, Cat. no. 4368813) was used to synthesize first-strand complementary (c)DNA from total RNA. According to the manufacturer’s instructions, 2.0 µL of 10× RT buffer, 0.8 µL of 25× dNTP Mix, 2.0 µL of 10 RT Random Primer, 1.0 µL of MultiScribe Reverse Transcriptase and 4.2 µL of nuclease-free water were mixed gently on ice as 2× RT master mix, then mixed with 10 µL of RNA in each tube. The thermal cycler was used to perform reverse transcription (RT) with the following conditions, 25 °C for 10 min, 37 °C for 120 min, 85 °C for 5 min and hold at 4 °C. A RT-qPCR was performed using LightCycler^®^ 480 SYBR Green I Master (Roche Diagnostics, Indianapolis, IN) on a Bio-Rad iCycler using GAPDH as the control, and data were analysed using the 2^−ΔΔCt^ method. Primer sequences used to perform the real-time qPCR are listed in Table 1.

**Table 1.** Sequence of primers for the human *MMP-1*, *COL1A1* and *GADPH* genes.

Gene	Forward (F) and reverse (R) primers
*MMP-1*	F: ATGAAGCAGCCCAGATGTGGAG
	R: TGGTCCACATCTGCTCTTGGCA
*COL1A1*	F: GATTCCCTGGACCTAAAGGTGC
	R: AGCCTCTCCATCTTTGCCAGCA
*GAPDH*	F: AGCCACATCGCTCAGACAC
	R: GCCCAATACGACCAAATCC

### Statistical analysis

All results are presented as the mean ± standard deviation (SD) of values obtained from three or more independent experimental replications. Statistical significance was determined by a one-way analysis of variance (ANOVA) with the *post hoc* Student–Newman–Keuls method. A *p* value of <0.05 was regarded as statistically significant.

## Results

### Chemical components of *P. calleryana* in different months

#### Extraction rates of PC in different months

To comprehensively understand the phytochemicals of PC including their chemical diversity and seasonal transmission, our workflow encompassed the collection of PC twigs and leaves from the Matsu Islands, Taiwan from 2018 to 2020. Samples underwent two rounds of extraction using either 95% EtOH or H_2_O, with a sample-to-solvent volume ratio of 1:10. Subsequently, extraction rates of all experimental samples were then calculated. Overall, extraction rate indices for PC leaves were higher than those of twigs and ranged 12.22–19.44% for the EtOH extract (PC-LE) and 14.71–28.74% for the H_2_O extract (PC-LH). Rates for H_2_O extracts of twigs (PC-SH) were 1.13–4.15%, whereas EtOH extracts of twigs (PC-SE) showed lower rates of 0.91–3.65% (Table 2). Extraction rates for PC twigs varied across different months and years, with EtOH extraction rates generally lower than water extraction rates. While no clear seasonal pattern emerged, there were fluctuations throughout the year. For instance, in January 2020, the EtOH extraction rate for twigs (PC-SE01) was 1.80%, whereas the water extraction rate (PC-SH01) was 3.63%. In contrast, in March 2020, the EtOH extraction rate (PC-SE03) had dropped to 0.91%, while the water extraction rate (PC-SH03) was 2.08%.

**Table 2.** Extraction rates of *Pyrus calleryana* (PC) samples from different months.

Month	Year	Part	Solvent	Label	Extraction rate (%)
January	2020	Twigs	95% EtOH	PC-SE01	1.80
			H_2_O	PC-SH01	3.63
February	2020	Twigs	95% EtOH	PC-SE02	2.17
			H_2_O	PC-SH02	2.92
March	2020	Twigs	95% EtOH	PC-SE03	0.91
			H_2_O	PC-SH03	2.08
		Leaf	95% EtOH	PC-LE03	17.93
			H_2_O	PC-LH03	27.62
April	2019	Twigs	95% EtOH	PC-SE04	1.00
			H_2_O	PC-SH04	2.77
		Leaf	95% EtOH	PC-LE04	17.18
			H_2_O	PC-LH04	19.65
May	2018	Twigs	95% EtOH	PC-SE05	2.79
			H_2_O	PC-SH05	2.67
		Leaf	95% EtOH	PC-LE05	16.67
			H_2_O	PC-LH05	20.07
June	2018	Twigs	95% EtOH	PC-SE06	2.71
			H_2_O	PC-SH06	3.52
		Leaf	95% EtOH	PC-LE06	12.22
			H_2_O	PC-LH06	14.97
July	2018	Twigs	95% EtOH	PC-SE07	3.65
			H_2_O	PC-SH07	4.15
		Leaf	95% EtOH	PC-LE07	18.59
			H_2_O	PC-LH07	23.63
August	2018	Twigs	95% EtOH	PC-SE08	1.87
			H_2_O	PC-SH08	3.13
		Leaf	95% EtOH	PC-LE08	12.45
			H_2_O	PC-LH08	14.71
September	2019	Twigs	95% EtOH	PC-SE09	1.02
			H_2_O	PC-SH09	3.40
		Leaf	95% EtOH	PC-LE09	19.44
			H_2_O	PC-LH09	28.74
October	2018	Twigs	95% EtOH	PC-SE10	2.38
			H_2_O	PC-SH10	1.13
		Leaf	95% EtOH	PC-LE10	19.08
			H_2_O	PC-LH10	19.00
November	2019	Twigs	95% EtOH	PC-SE11	2.44
			H_2_O	PC-SH11	3.73
December	2019	Twigs	95% EtOH	PC-SE12	2.71
			H_2_O	PC-SH12	2.88

Similarly, extraction rates for PC leaves exhibited significant variation across months and years, with both EtOH and water extraction rates notably higher compared to twigs. Notably, there seemed to be a trend of higher extraction rates during warmer months, such as May to July, compared to colder months like November to February. For example, in March 2020, the EtOH extraction rate for leaves (PC-LE03) surged to 17.93%, while the water extraction rate (PC-LH03) peaked at 27.62%, marking considerable increases from previous months.

### Total phenolic contents of PC extracts

Total phenolic contents of PC extracts from different months were analysed using the Folin–Ciocalteu reagent, with gallic acid as the standard (Chang and Lin 2012). Results of the analysis are expressed in grams of GAE per 100 g of dried material. Table 3 illustrates the total phenolic contents of PC extracts; overall, phenolic contents of twig extracts (PC-SE and PC-SH) of PC were higher than those of leaf extracts (PC-LE and PC-LH). Among them, the PC-SH water-extracted twig extract had the highest average phenolic contents among the four types of PC extracts and ranged 16.88 to 37.81 g GAE/100 g dry material (Table 3). There were notable variations in PC-SE and PC-SH across different months. Both PC-SE and PC-SH exhibited an increasing trend from January to June, reaching a peak in July, followed by a decline thereafter. On the other hand, PC-LE and PC-LH showed fluctuations from January to April, followed by relatively stable levels from May to August, and further fluctuations from September to December.

**Table 3.** Total phenolic contents of *Pyrus calleryana* (PC) extracts.

	Phenolic content (g GAE/100 g)			
Month	PC-SE	PC-SH	PC-LE	PC-LH
January	19.87 ± 0.10	27.88 ± 0.12	–	–
February	25.51 ± 0.07	36.25 ± 0.06	–	–
March	20.55 ± 0.04	32.08 ± 0.18	24.20 ± 0.29	25.10 ± 0.07
April	24.86 ± 0.09	35.98 ± 0.38	7.69 ± 0.02	9.13 ± 0.00
May	20.46 ± 0.06	16.88 ± 0.08	20.25 ± 0.02	21.81 ± 0.05
June	25.59 ± 0.12	34.69 ± 0.20	17.13 ± 0.08	19.43 ± 0.05
July	27.39 ± 0.12	24.34 ± 0.29	17.81 ± 0.05	15.79 ± 0.09
August	20.89 ± 0.32	36.20 ± 0.32	15.43 ± 0.13	15.43 ± 0.28
September	19.24 ± 0.10	33.00 ± 0.29	17.26 ± 0.23	21.98 ± 0.26
October	36.45 ± 0.19	22.92 ± 0.11	12.72 ± 0.06	17.42 ± 0.22
November	24.17 ± 0.07	28.32 ± 0.04	–	–
December	30.62 ± 0.17	37.81 ± 0.31	–	–

Sample concentration: 100 μg/mL. GAE: gallic acid equivalents (3.125, 6.25, 12.5, 25 and 50 μg/mL), *R*^2^ > 0.99.

### Total triterpenoid contents of PC extracts

A vanillin–glacial acetic acid–HClO_4_ colorimetric system was utilized to analyse total triterpene contents in different months’ extracts of Callery pear. Oleanolic acid was used as the standard for constructing the calibration curve. Results are expressed in grams of OAEs per 100 g of dried extract (Xu and Chang 2009). Table 4 displays total triterpenoid contents of PC extracts. Outcomes revealed that triterpene contents in twig extracts of Callery pear (PC-SE and PC-SH) were higher than those of leaf extracts (PC-LE and PC-LH), and 95% EtOH extracts contained more triterpenes than did water extracts. Among the four categories of Callery pear extracts, the PC-SE twig extract obtained from the 95% EtOH extraction exhibited the highest average triterpene contents that ranged 40.68–74.07 g OAE/100 g (Table 4). While the levels of PC-SE and PC-SH remain relatively consistent across different months, PC-LE and PC-LH showed more significant variations. PC-LE and PC-LH exhibited decreasing trends from January to April, followed by increases from May to August, and decreases from September to December.

**Table 4.** Total triterpenoid contents of *Pyrus calleryana* (PC) extracts.

	Triterpenoid content (g OAE/100 g)			
Month	PC-SE	PC-SH	PC-LE	PC-LH
January	35.30 ± 0.60	33.29 ± 0.68	–	–
February	45.63 ± 0.22	49.48 ± 0.07	–	–
March	48.94 ± 0.22	35.97 ± 0.03	21.75 ± 0.37	22.99 ± 0.10
April	46.34 ± 0.66	47.62 ± 1.26	11.42 ± 0.17	4.89 ± 0.11
May	64.34 ± 3.66	25.52 ± 0.58	13.06 ± 0.21	8.58 ± 0.19
June	52.02 ± 0.52	46.70 ± 0.39	14.52 ± 0.39	8.16 ± 0.20
July	66.31 ± 1.06	29.90 ± 0.49	20.26 ± 0.56	8.41 ± 0.40
August	60.00 ± 1.66	48.95 ± 0.75	16.63 ± 0.37	10.83 ± 0.58
September	40.68 ± 0.63	33.84 ± 0.26	27.86 ± 0.51	10.80 ± 0.73
October	74.07 ± 0.73	32.23 ± 1.73	21.38 ± 0.13	10.58 ± 0.21
November	47.19 ± 0.57	41.61 ± 0.40	–	–
December	50.56 ± 0.26	46.20 ± 0.57	–	–

Sample concentration: 100 μg/mL. OAE: oleanolic acid equivalents (3.125, 6.25, 12.5, 25, 50 and 100 μg/mL), *R*^2^ > 0.99.

### DPPH radical-scavenging activities of PC extracts

DPPH is a stable radical that is used in a popular method for screening the free radical-scavenging ability of compounds or antioxidant activities of plant extracts. Table 5 presents DPPH radical-scavenging activities of PC extracts. The DPPH free radical-scavenging abilities of the twig extracts (PC-SE and PC-SH) of PC were better than those of leaf extracts (PC-LE and PC-LH). DPPH free radical-scavenging rates of twig extracts obtained from water extraction and 95% EtOH extraction from PC were both >85% (Table 5). Scavenging activities of PC-SE and PC-SH showed minimal variations across different months, whereas PC-LE and PC-LH demonstrated more significant fluctuations. Generally, the DPPH radical-scavenging activity increased with higher levels of total phenolic and triterpenoid contents. However, exceptions existed, such as in April, when PC-LE and PC-LH exhibited lower contents but higher scavenging activities.

**Table 5.** DPPH radical-scavenging activities of *Pyrus calleryana* (PC) extracts.

	Scavenging activity (% ±SD)			
Month	PC-SE	PC-SH	PC-LE	PC-LH
January	90.53 ± 0.11	85.47 ± 0.35	–	–
February	94.19 ± 0.14	92.13 ± 0.18	–	–
March	92.20 ± 0.07	86.16 ± 0.07	90.34 ± 0.07	91.33 ± 0.23
April	91.64 ± 0.13	96.02 ± 0.48	22.21 ± 1.00	44.57 ± 1.23
May	94.56 ± 0.05	92.19 ± 0.06	71.52 ± 0.53	94.40 ± 0.26
June	94.35 ± 0.10	98.77 ± 1.25	60.01 ± 1.44	89.41 ± 1.18
July	95.39 ± 0.11	93.86 ± 0.29	64.31 ± 0.81	87.15 ± 0.82
August	94.41 ± 0.11	95.72 ± 0.22	52.94 ± 0.29	82.07 ± 2.22
September	86.57 ± 0.98	92.98 ± 0.94	56.73 ± 1.00	93.54 ± 0.23
October	94.61 ± 0.07	91.10 ± 1.26	37.49 ± 2.52	84.26 ± 0.24
November	93.82 ± 0.18	91.68 ± 0.38	–	–
December	94.70 ± 0.07	88.72 ± 0.47	–	–

SD: standard deviation.

Sample concentration: 100 μg/mL; scavenging rate of the solvent control: 0.00% ± 1.68%; scavenging rate of the positive control (trolox 60 μM): 93.62% ± 0.31%.

### LC–MS/MS-based analysis of PC extracts

#### Principal component analysis (PCA) of PC extracts

**Figure 2.**
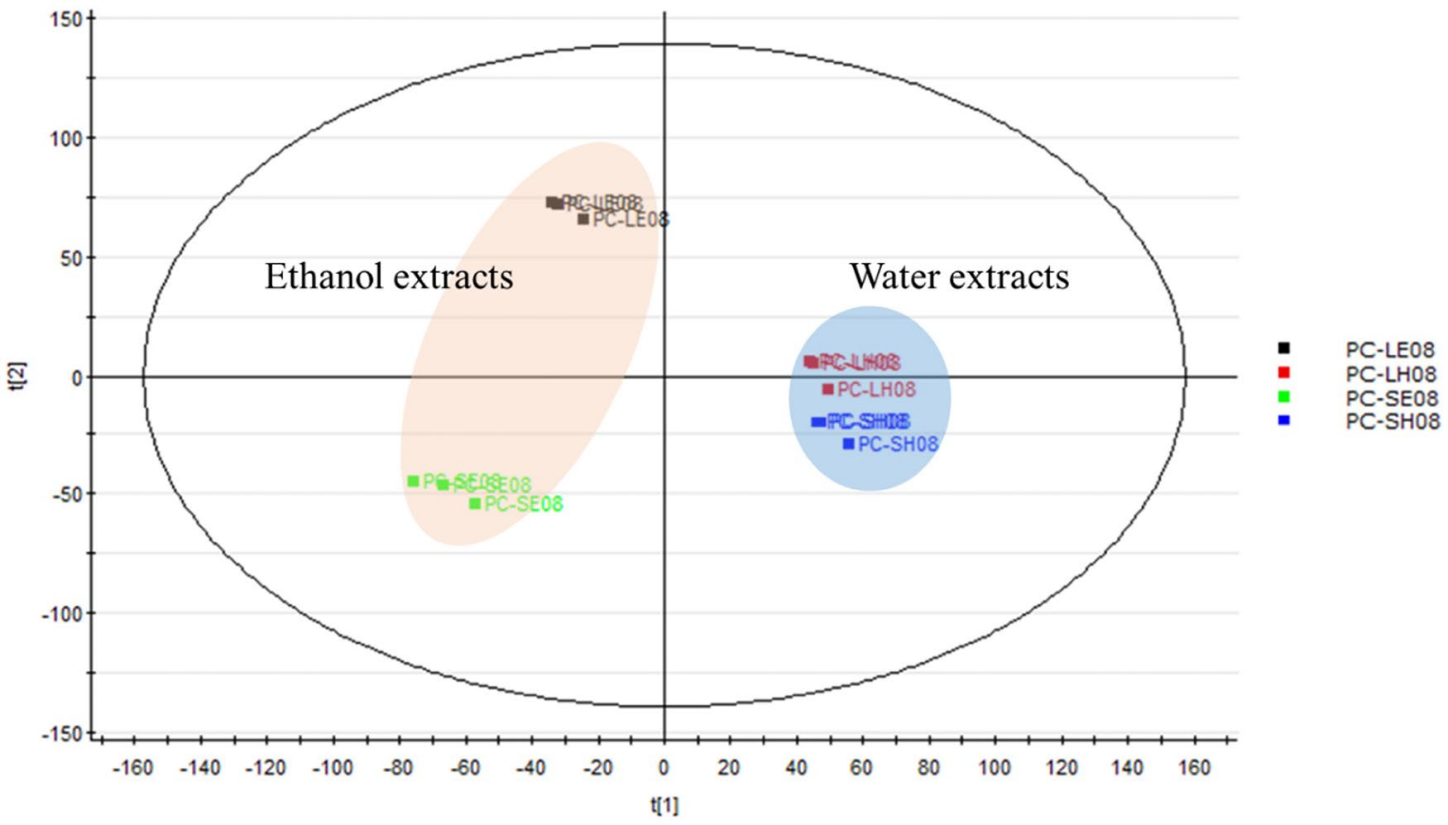
Principal component analysis of four *Pyrus calleryana* (PC) extracts from August. PC-LE08: 95% ethanol leaf extract of PC; PC-LH08: water leaf extract of PC; PC-SE08: 95% ethanol twig extract of PC; PC-SH08: water twig extract of PC.

A PCA is one of the most important and powerful methods in chemometrics. In addition, it can also identify effects of technical variations in the analysis of metabolic profiles, which is crucial for data quality evaluation in metabolomic studies. Following pre-processing of LC–MS/MS data of PC extracts in August, the PCA allowed a descriptive evaluation of the sample distribution to assess data quality and identify natural groupings, patterns and outliers. Samples presented in the PCA score plot in Figure 2 show close clustering between data points for each time interval. This indicated good quality, stability, reliability and reproducibility of the analyses. The score plot displays the relationship between different extraction methods for twigs and leaves of PC. In our results, PCA models revealed different extraction method trend groupings for PC metabolites. The 95% EtOH-extracted PC samples (PC-SE08 and PC-LE08) and water-extracted PC samples (PC-SH08 and PC-LH08) were distributed in different regions on the left and right sides of the plot. This indicates significant differences in the compositions of the extracts when using different solvents. Water-extracted PC twig and leaf samples (PC-SH08 and PC-LH08) were clustered in a similar region, suggesting that their compositions were likely more similar. On the other hand, the two samples extracted with 95% EtOH (PC-SE08 and PC-LE08) were more widely spread apart, indicating greater differences in their compositions.

**Figure 3.**
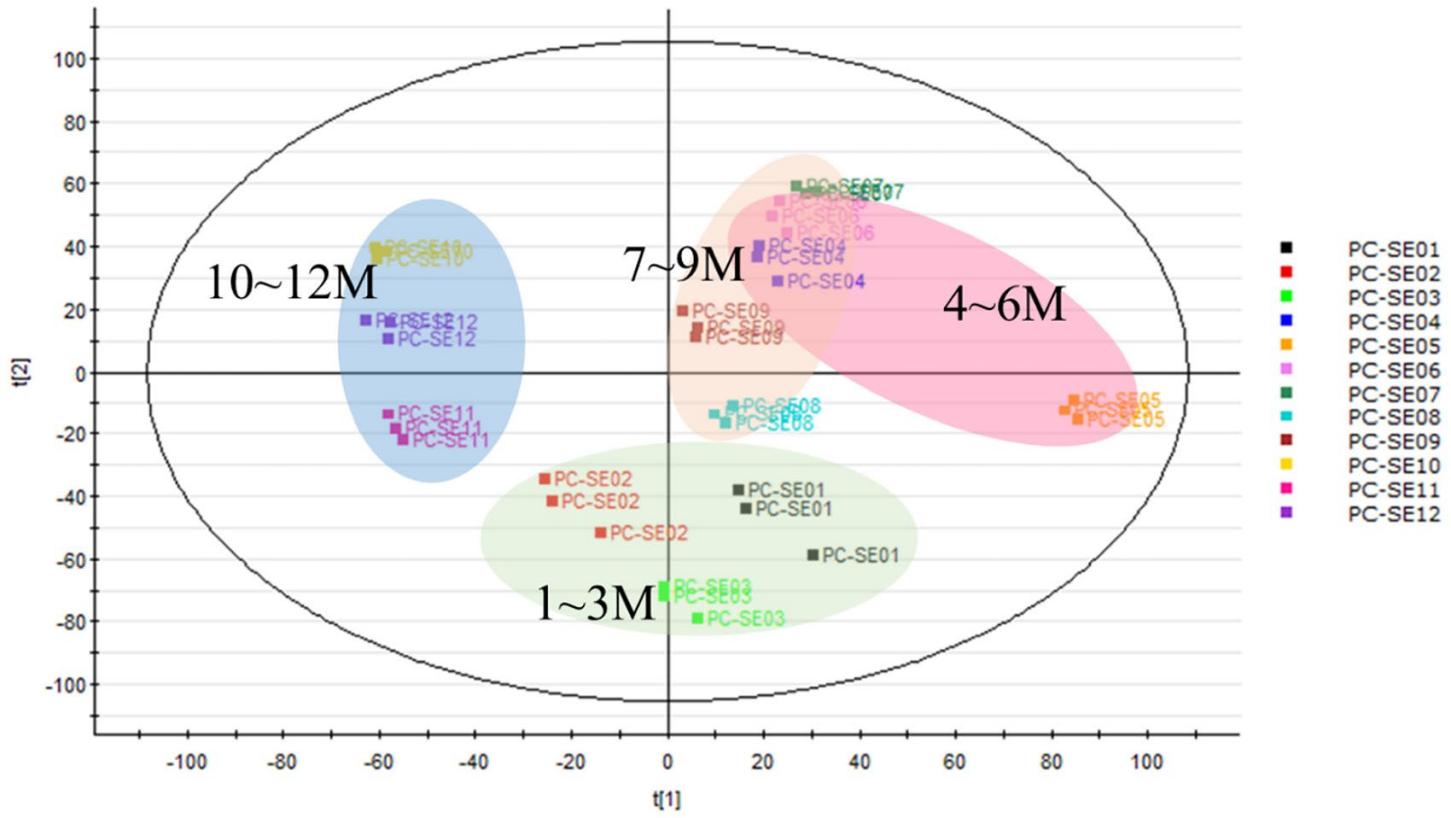
Principal component analysis of *Pyrus calleryana* (PC) ethanol twig (SE) extracts from different months. PC-SE01–12: 95% ethanol twig extract collected in different months (January–December).

Similarly, the PCA plot of an LC–MS/MS analysis of 95% EtOH-extracted twigs of PC samples collected in different months showed that samples from months closer together in time were clustered together, indicating that their compositions were more similar. For instance, EtOH-extracted twigs of samples from January to March (spring) were situated in the lower part of the plot. Samples from April to June (summer) and July to September (autumn) were positioned in the upper-right part of the plot, while samples from the months of October to December (winter) were situated in the left part of the plot (Figure 3).

### Exploring the metabolomic diversity of PC twigs using an MN approach

**Figure 4.**
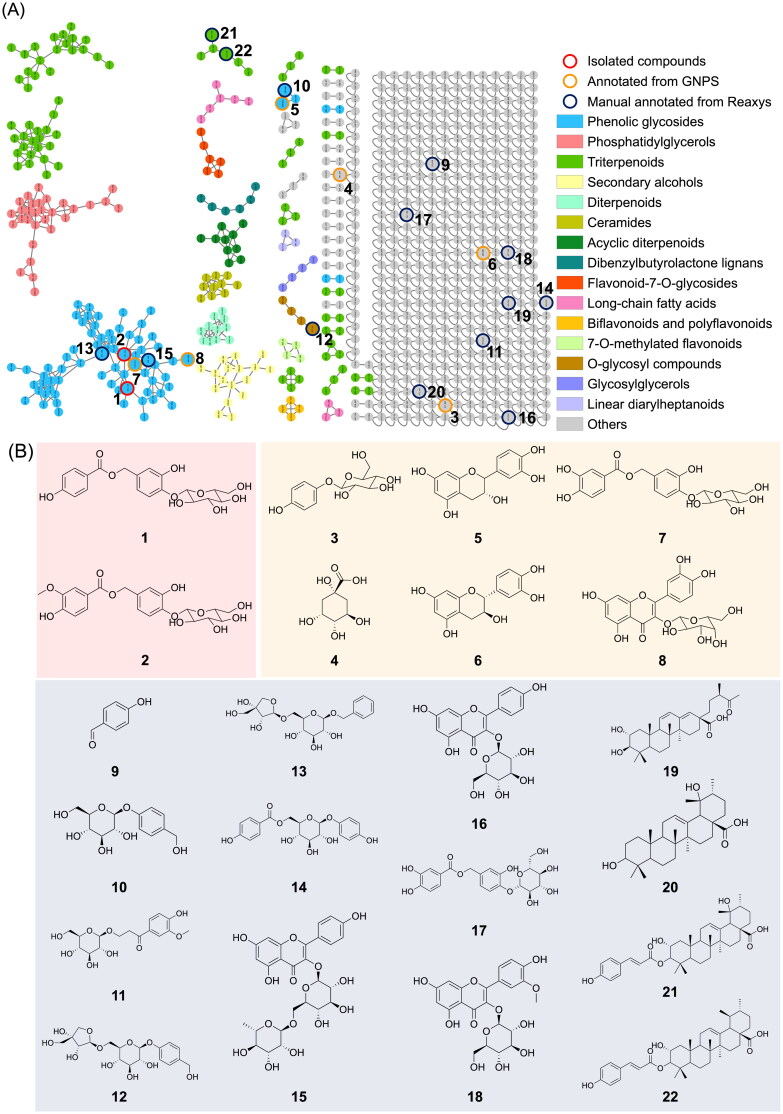
Molecular network of *Pyrus calleryana* (PC) extract in negative mode. (A) Chemicals classified according to ClassyFire parents. (B) Structures of isolates from the 95% ethanol (PC95E) extract and annotations from the GNPS library.

Ethanol extracts of PC twigs were prepared and subjected to an MS/MS analysis using LC-QToF. The collected MS/MS data were further processed on the GNPS MN platform, and then Cytoscape and MolNetEnhancer tools were applied to lay out the metabolomic profile. Putative chemical classifications were further analysed by ClassyFire software, indicating the presence of the majority of phenolic glycoside metabolites in the PC extract (Figure 4(A)). Moreover, constituents from the PC extract were putatively annotated using GNPS, the Reaxys database, and our in-house database matches, which led to the identification of 22 compounds, belonging to phenolic glycosides, triterpenoids and others (Figure 4(B)).

**Figure 5.**
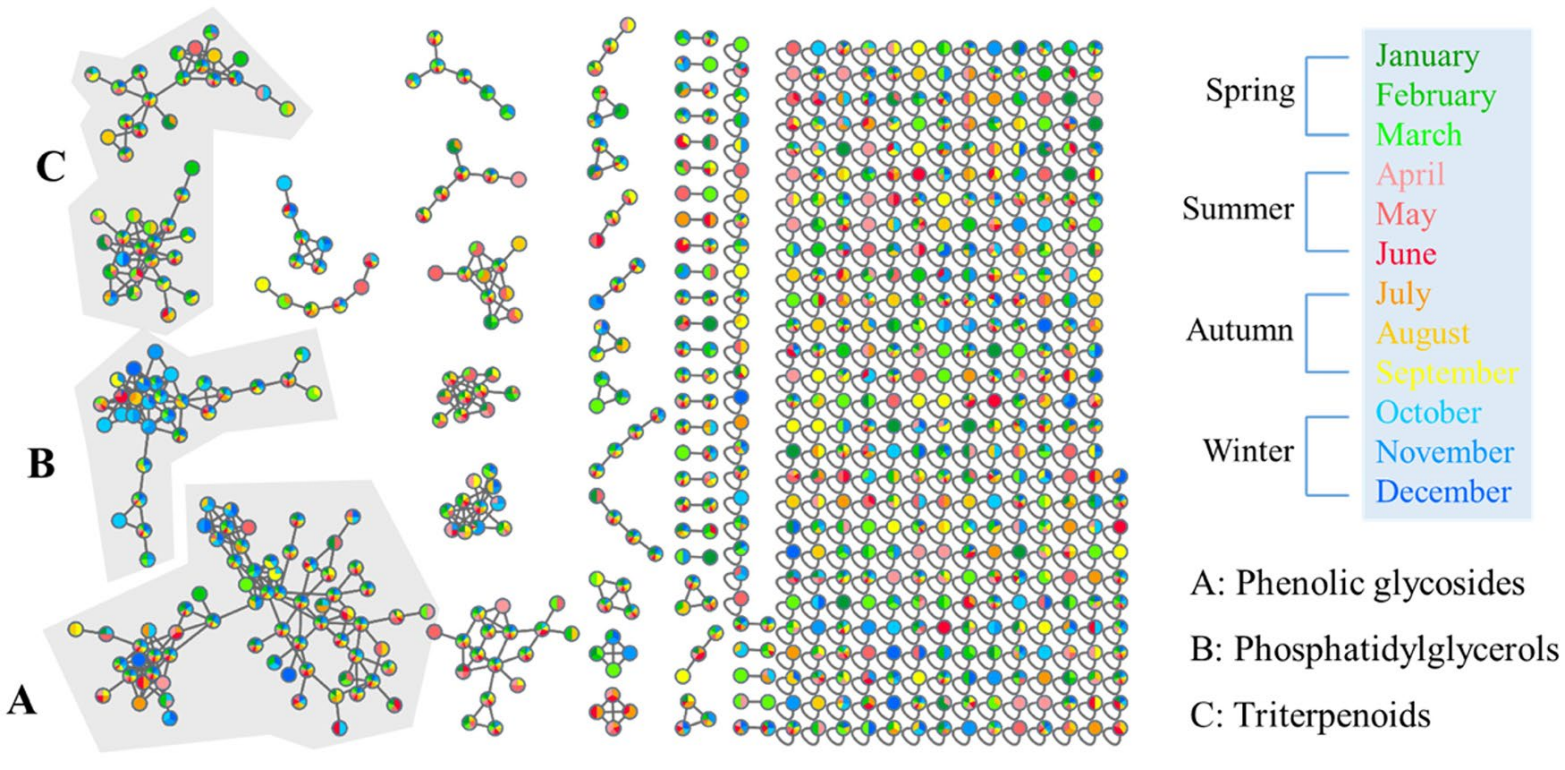
Molecular network of *Pyrus calleryana* (PC) ethanolic twig (SE) extracts from different months coloured according to month. PC-SE extracts were analysed using LC–MS/MS, and chromatograms were exported to GNPS for molecular networking. Nodes in the network are coloured according to the mean precursor ion intensity of extracts from each month. Molecular families A (phenolic glycosides), B (phosphatidylglycerols), and C (triterpenoids) are highlighted.

Examining the larger molecular clusters within the network diagram (Figure 5), categories such as phenolic glycosides, phosphatidylglycerols and triterpenoids were identified. Observing the colour distribution of nodes corresponding to different months, it was noted that the colour distribution was relatively uniform across months within the clusters of phenolic glycosides and triterpenoids. This indicates the presence of these compound types throughout the year in the 95% EtOH extracts of PC twigs. For clusters of phosphatidylglycerols, a higher proportion of blue tones (representing the months of October to December) was observed. This suggests a higher distribution of this compound type during colder months.

### Effects of isolates from PC on wound healing

#### Identification of compounds

Phenolic compounds that possess antioxidant properties play significant and pivotal roles in the realm of food research. This involves the fact that phenolic compounds exhibit commendable potential in preventing and/or treating a wide array of diseases, including but not limited to cancer, diabetes, cardiovascular illnesses and neurodegenerative conditions. Our research aimed to explore the bioactive compounds derived from PC with a focus on their potential for wound-healing applications. To achieve this, we employed a bio-guided approach to meticulously select fractions that harboured bioactive compounds of interest. Through our diligent efforts, we successfully isolated two noteworthy compounds from the ethanolic extract of PC twigs: 3′-hydroxylbenzyl-4-hydroxybenzoate-4′-*O*-β-glucopyranoside (**1**) and vanilloylcalleryanin (**2**).

### Assessment of the roles of compounds in cell proliferation

**Figure 6.**
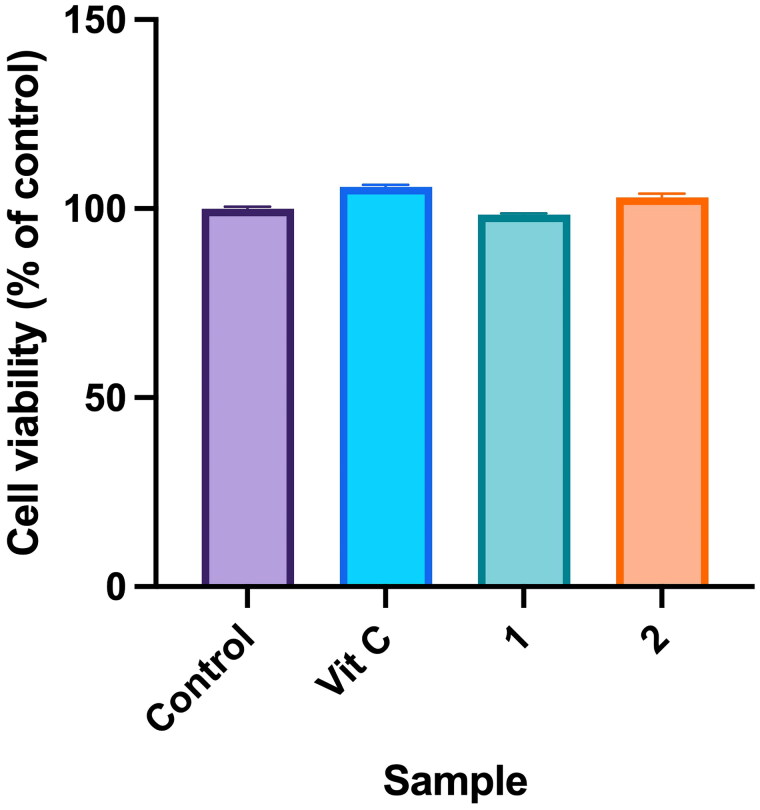
Effects of compounds 3′-hydroxylbenzyl-4-hydroxybenzoate-4′-*O*-β-glucopyranoside (**1**) and vanilloylcalleryanin (**2**) on cell viability of WS-1 cells. WS-1 cells were seeded in a 48-well plate and treated with compounds (100 μM) isolated from *Pyrus calleryana* for 24 h. Vitamin C (ascorbic acid, 100 μM) was used as a positive control. Cell viability was measured by an MTT assay. Results are expressed as a ratio relative to the control. Each determination was performed in triplicate, and values are presented as the mean ± standard deviation.

Fibroblasts play a crucial role in the wound-healing process, which is a complex and highly orchestrated sequence of events that aims to restore tissue integrity and functionality following an injury. During the proliferative phase, which typically begins a few days after injury, fibroblasts become highly active. Their primary functions include ECM production, collagen synthesis, wound contraction, angiogenesis promotion, immune regulation and matrix remodelling (Pillouer-Prost 2003). To assess the cytotoxicity of compounds isolated from PC towards human dermal fibroblast (WS-1) cells, a cell viability assay using the MTT method was conducted. Results showed that the cell viability of compounds **1** and **2** was 98.40% ± 0.37% and 103.02% ± 0.94%, respectively. There were no significant differences compared to the control group, indicating that neither **1** nor **2** exhibited cytotoxicity (Figure 6).

### Evaluation of the role of isolates in cell migration

**Figure 7.**
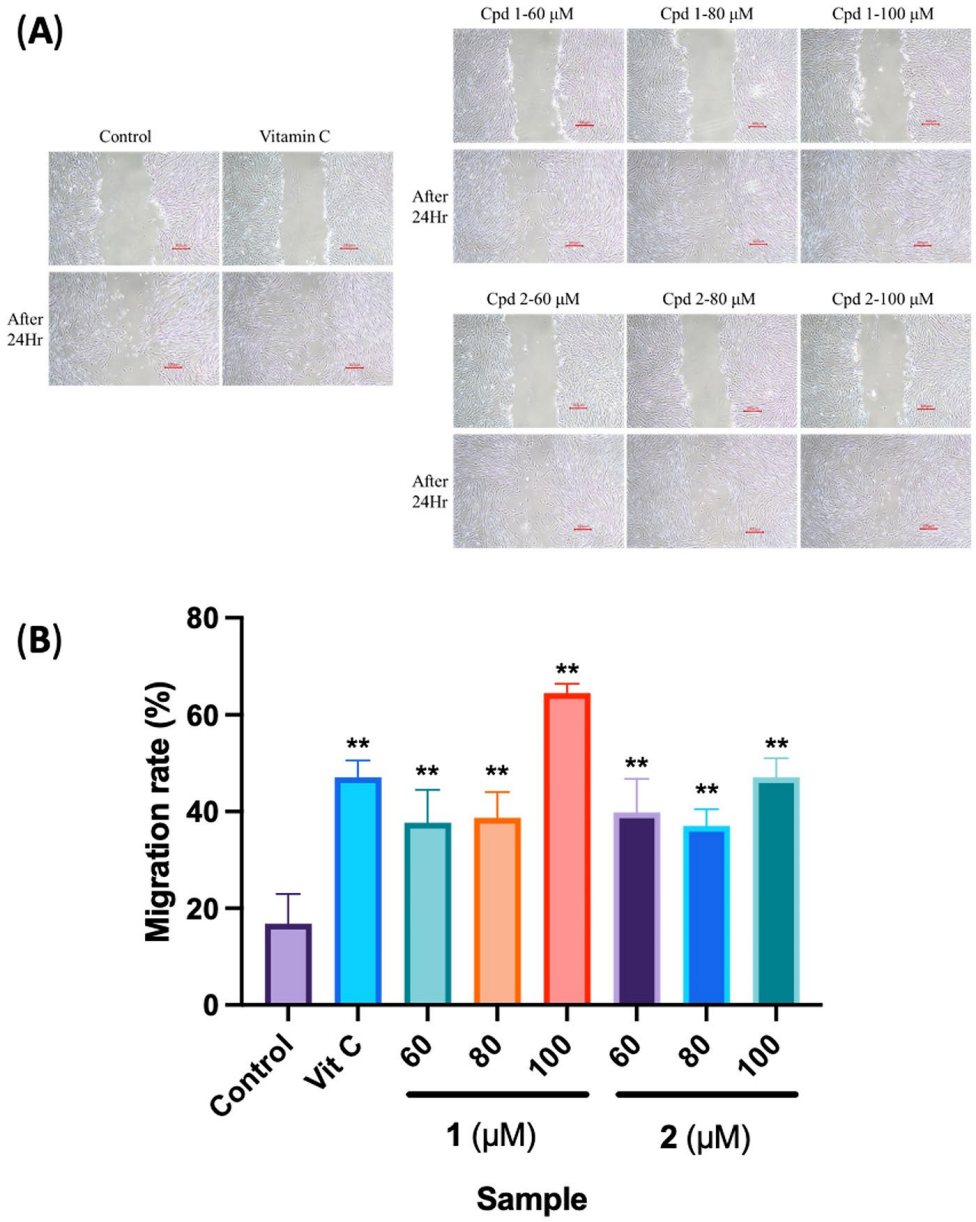
Effects of compounds 3′-hydroxylbenzyl-4-hydroxybenzoate-4′-*O*-β-glucopyranoside (**1**) and vanilloylcalleryanin (**2**) on the cell migration of WS-1 cells. (A) Wound-healing assay of WS-1 cells incubated with different concentrations of the 95% ethanolic extract of *Pyrus calleryana* (PC95E) compounds. (B) Cells were treated with compounds (60, 80 and 100 μM) isolated from PC for 24 h. Vitamin C (ascorbic acid, 100 μM) was used as a positive control. Photos were taken before and after treatment, and scratched areas were measured using ImageJ software (Bethesda, MD). Cell migration rates were calculated. Each determination was performed in triplicate, and values are presented as the mean ± standard deviation. **p < .01 vs. the control group.

To estimate the wound reepithelialization potential of compounds and to assess quality control of the assay, a standard is required. Several growth factors and cytokines were reported to directly or indirectly affect fibroblast motility. Ascorbic acid is involved in all phases of wound healing. In the inflammatory phase, it is required for neutrophil apoptosis and clearance. During the proliferative phase, ascorbic acid contributes to the synthesis, maturation, secretion and degradation of collagen; so ascorbic acid was used as a positive control (Ravi et al. 2023). Effects of the two compounds on fibroblast migration and proliferation were investigated under established conditions. Results showed that isolated compounds **1** and **2** of PC extracts significantly enhanced the migration of human dermal fibroblasts compared to the control group (Figure 7(A)). Through a software analysis, calculating the scratch area and migration rate, the cell-migration rate of the control group was 16.86% ± 6.13%, and that of the positive control group was 47.10% ± 3.47%. For compound **1** at 60, 80 and 100 μM, cell-migration rates were 37.73% ± 6.76%, 38.69% ± 5.31% and 64.49% ± 1.92%, respectively, and for compound **2** at 60, 80 and 100 μM, respective cell-migration rates were 39.84% ± 6.95%, 37.05% ± 3.43% and 47.09% ± 3.95% (Figure 7(B)). Both compounds **1** and **2** exhibited the best promotion of migration in the 100 μM group, with compound **1** at 100 μM showing the highest migration rate.

### Effects of compounds on expression of MMP1 and COL1A1

**Figure 8.**
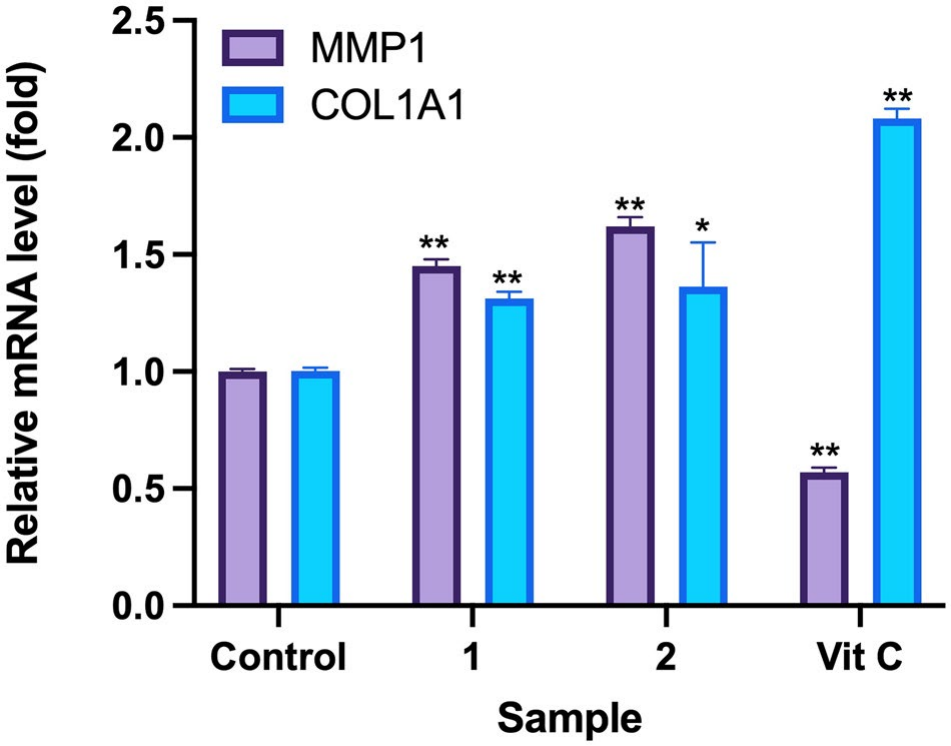
Effects of compounds 3′-hydroxylbenzyl-4-hydroxybenzoate-4′-O-droxylbenzyl-4-hy (**1**) and vanilloylcalleryanin (**2**) on gene expression by WS-1 cells. WS-1 cells were seeded in 6-cm dishes and treated with compounds (100 μM) isolated from *Pyrus calleryana* for 24 h. Vitamin C (ascorbic acid, 100 μM) was used as a positive control. RNA extraction, reverse transcription and a real-time PCR were performed. Each determination was performed in triplicate, and values are presented as the mean ± standard deviation. *p < .05 and **p < .01 vs. the control group.

An RT-qPCR assay was utilized to investigate the impact of Callery pear compounds on expression of the wound healing-related genes *MMP1* and *COL1A1* in human dermal fibroblasts (WS-1). These compounds were examined at a concentration of 100 μM, with ascorbic acid as the positive control group. Results revealed significant alterations in expression of the *MMP1* and *COL1A1* genes. Specifically, for compound **1**, the expression level of the *MMP1* gene was 1.45 ± 0.03 times, while expression level of the *COL1A1* gene was 1.31 ± 0.03 times. For compound **2**, the expression level of the *MMP1* gene was 1.62 ± 0.04 times, and expression level of the *COL1A1* gene was 1.36 ± 0.19 times. Expression levels of the *MMP1* and *COL1A1* genes in the positive control group were 0.88 ± 0.01 and 1.39 ± 0.08 times, respectively (Figure 8).

Furthermore, analysis of *MMP1* and *COL1A1* secretion levels by WS-1 fibroblast cells demonstrated increased secretion of ECM components associated with wound healing upon treatment with either compound **1** or **2** at a concentration of 100 μM. This effect was notably distinct from observations in the negative control groups.

## Discussion

The chemical evaluation in regard to extraction rates, and total phenolic and total triterpenoid contents of PC extracts suggest that there were significant variations in the chemical composition of PC extracts among different months and between different plant parts (twigs and leaves), as well as between extraction solvents (95% EtOH and H_2_O). Several important points emerged from the results.

The DPPH radical-scavenging assay results suggested that PC extracts, especially from twigs, possessed strong antioxidant properties. Upon reviewing bioactivities of plant species belonging to the *Pyrus* genus, it is conspicuously apparent that investigations pertaining to antioxidant properties, or the scavenging of free radicals, exhibited robust associations with phenolic compounds (Ibrahim and Hammoudi 2020). Phenolic compounds have repeatedly been implicated as natural antioxidants in fruits, vegetables and other plants. For instance, compounds like caffeic acid, ferulic acid and vanillic acid are extensively found throughout the plant kingdom. Protocatechuoylcalleryanin, a compound isolated from the EtOH extract of PC, exhibited a notable free radical-scavenging capacity, demonstrating antioxidant activity in the previous literature (Challice et al. 1980). These antioxidants could potentially have health benefits and applications in the food and pharmaceutical industries.

Variations observed in the composition of PC extracts across different solvents (ethanolic and water) and plant parts (stem and leaf) indicate dynamic changes in phenolic and triterpenoid contents throughout the year. Levels of phenolic and triterpenoid compounds fluctuated seasonally, with some months showing higher concentrations than others. Additionally, relationships between extract compositions and DPPH radical-scavenging activities are complex, with fluctuations in scavenging activities not always directly correlated with phenolic and triterpenoid levels. These findings highlight the importance of considering multiple factors when assessing the antioxidant properties of plant extracts. Further research is needed to elucidate the underlying mechanisms driving these variations and their implications for potential therapeutic applications of PC extracts.

Exploring the metabolomic diversity of PC twigs using a GNPS MN approach suggested a higher distribution of phosphatidylglycerols during colder months (October to December). Phosphatidylglycerols are composed of a structure in which saturated or unsaturated fatty acids are connected to an l-glycerol 3-phosphate backbone. In plants, these compounds are mainly found in thylakoid membranes and are associated with photosynthesis (Hagio et al. 2002). Previous research indicated that the proportion of phosphatidylglycerols in plants can change with growth temperature or season. For instance, plants cultivated in colder environments tend to exhibit elevated proportions of unsaturated fatty acids, such as linolenic acid (18:3), in phosphatidylglycerol compounds. This adaptation of lipid saturation levels in membranes aids in preserving membrane fluidity under low temperatures (Jahed et al. 2023). During the process of phosphatidylglycerol synthesis, two primary fatty acids, palmitic acid (16:0) and oleic acid (18:1), are synthesized in the form of acyl carrier protein-bound compounds. They are attached to the main backbone through the action of glycerol-3-phosphate acyltransferase (GPAT) and subsequently undergo desaturation by fatty acid desaturase to reduce their saturation levels. Changes in GPAT selectivity and the activity of fatty acid desaturase under low-temperature conditions might contribute to the decrease in saturation levels of phosphatidylglycerols (Xiao et al. 2022).

Two phenolic glycoside compounds, 3′-hydroxylbenzyl-4-hydroxybenzoate-4′-*O*-β-glucopyranoside (**1**) and vanilloylcalleryanin (**2**), were isolated in the present study. These compounds are classified as phenolic glycosides and are constituents of calleryanin. Notably, calleryanin, also known as 3,4-dihydroxybenzyl alcohol 4-glucoside, was initially isolated from PC in 1968 (Challice and Williams 1968), marking the first discovery of this unique chemical structure in the realm of natural compounds. Calleryanin is known for its potential antioxidant properties, which means it may help protect cells from oxidative damage caused by free radicals (Nassar et al. 2011). Until now more than 20 phenolic glycosides have been isolated from *Pyrus* spp. (Bilia et al. 1994; Nassar et al. 2011; Li et al. 2016; Zhao et al. 2016). These compounds have various bioactivities, such as hepatoprotective (Basnet et al. 1996; Xiang et al. 2001), anti-inflammatory (Hammer et al. 2008), cytotoxic (Lee et al. 2007), antiprotozoal (Kirmizibekmez et al. 2004) and antioxidative actions (Nassar et al. 2011).

The cytotoxicity of compounds isolated from PC towards human dermal fibroblast (WS-1) cells was evaluated with an MTT assay. Based on the presented results, it is evident that neither of the tested compounds exhibited cytotoxic effects. Cell viability percentages of compounds **1** and **2** were closely aligned with the control group, strongly suggesting that these compounds are unlikely to cause harm to fibroblast cells. Furthermore, wound-healing assay results implied that higher concentrations of compounds **1** and **2** may yield more-significant benefits in terms of promoting cell migration. The study found that at a concentration of 100 μM, both compounds substantially enhanced the migration of human dermal fibroblasts. This suggests a dose-dependent response, where increasing the concentration of these compounds may lead to even more-pronounced effects on cell migration, a pivotal aspect of the wound-healing process. Additionally, the study’s assessment of ECM components, specifically *MMP1* and *COL1A1*, which are closely linked to wound healing, provided valuable insights. Collagen, represented by the *COL1A1* gene in our study, is a crucial protein in the ECM that provides mechanical support to tissues and is essential for tissue repair. An increase in *COL1A1* secretion implies improved collagen synthesis, which is essential for the formation of strong and functional scar tissue during wound healing (Almeida et al. 2015; Reilly and Lozano 2021). Similarly, *MMP1* is an enzyme that plays a pivotal role in tissue remodelling by breaking down old or damaged collagen. Its increased secretion indicates an active process of ECM remodelling, which is a fundamental aspect of wound healing, as it allows for the replacement of damaged tissues with new, healthy tissues (Stevens and Page-McCaw 2012; Mu et al. 2013). Treatment with compounds **1** and **2** at a concentration of 100 μM resulted in notable increases in the secretion of these critical ECM components. This signifies that these compounds have the potential to bolster the production of vital proteins involved in tissue remodelling and the overall wound-healing process. In conclusion, the study’s findings underscore the safety of compounds **1** and **2** with respect to fibroblast cells and suggest a dose-dependent relationship between their concentrations and their ability to promote cell migration. Furthermore, the observed increases in secretion of ECM components at a 100 μM concentration indicate the potential of these compounds to play a role in tissue remodelling and wound healing. Further research is warranted to elucidate the mechanisms underlying these effects and explore the practical applications of these compounds in wound-healing therapies.

## Conclusions

We employed a GNPS approach to analyse the 95% EtOH extract of twigs from PC using LC–MS/MS. Results of our analysis revealed a high content of phenolic glycosides in the ethanolic extract of twigs. Subsequently, we isolated and purified two distinct phenolic glycoside compounds: 3′-hydroxylbenzyl-4-hydroxybenzoate-4′-*O*-β-glucopyranoside (**1**) and vanilloylcalleryanin (**2**). The present study conducted the first investigation into the impacts of two specific compounds, 3′-hydroxylbenzyl-4-hydroxybenzoate-4′-*O*-β-glucopyranoside (**1**) and vanilloylcalleryanin (**2**), on human dermal fibroblasts. These compounds displayed remarkable capabilities in enhancing the wound-healing process, as demonstrated by their capacity to elevate expressions of the *MMP1* and *COL1A1* genes. Our findings, based on a combination of cell-scratch tests and gene-expression analyses, suggested that these two compounds hold promise for enhancing wound healing. Further research is necessary to fully understand their practical applications in wound healing.

## Data Availability

Data will be made available upon reasonable request.
